# Genomic Characterization of Multidrug-Resistant *Escherichia coli* from Bovine Mastitis and Therapeutic Evaluation of Thanatin Combined with Gallium Nitrate

**DOI:** 10.3390/microorganisms14071538

**Published:** 2026-07-14

**Authors:** Zhuo Li, Yuting Zhang, Danrui Bu, Zhao Liu, Jiahua He, Tingting Wan, Yan Lu, Xiaoye Liu

**Affiliations:** 1 College of Veterinary Medicine, Beijing University of Agriculture, No. 7 Beinong Road, Changping, Beijing 102206, China; lizhuo1@bua.edu.cn (Z.L.); yutingzhang@bua.edu.cn (Y.Z.); 202420322052@bua.edu.cn (D.B.); liuzhao@bua.edu.cn (Z.L.); wantingting@bua.edu.cn (T.W.); 2 Beijing Key Laboratory of Traditional Chinese Veterinary Medicine, Beijing University of Agriculture, No. 7 Beinong Road, Changping, Beijing 102206, China; jiahuahe@bua.edu.cn; 3 Beijing Traditional Chinese Veterinary Engineering Center, Beijing University of Agriculture, No. 7 Beinong Road, Changping, Beijing 102206, China

**Keywords:** bovine mastitis, *Escherichia* spp., multidrug resistance, combination therapy, thanatin, gallium nitrate

## Abstract

Bovine mastitis caused by multidrug-resistant (MDR) *Escherichia coli* remains a major challenge in the dairy industry due to recurrent infection, excessive lipopolysaccharide (LPS) release, and persistent mammary inflammation. Whole-genome sequencing (WGS) revealed that the clinical isolate was resistant to seven classes of antibiotics and exhibited a marked enrichment of iron uptake systems, which accounted for 27.14% of the 140 virulence genes identified. Upon this, we developed a targeted combination therapy using the iron mimetic gallium nitrate and the antimicrobial peptide thanatin (Tn). A rat model of mastitis was established to evaluate the in vivo therapeutic efficacy of the Tn-gallium nitrate combination. Our results demonstrated that the combination of gallium nitrate and Tn exerted a potent enhanced therapeutic effect in the rat mastitis model. This regimen significantly inhibited bacterial proliferation, neutralized endotoxin activity, and downregulated the expression of pro-inflammatory cytokines. Histopathological evaluation confirmed that the combination therapy effectively protected the alveolar structure and alleviated tissue damage, with a protective effect superior to that of ceftiofur (CEF), the first-line clinical drug. Collectively, our findings demonstrate the therapeutic potential of the combination of thanatin and gallium nitrate in a rat model of mastitis and provide experimental evidence supporting the development of a novel non-antibiotic therapeutic strategy for bovine mastitis caused by multidrug-resistant *E. coli*.

## 1. Introduction

Bovine mastitis is a prevalent disease in the dairy farming industry, which not only causes massive dairy product waste and severe economic losses but also directly impairs animal welfare and food safety [1,2,3]. Current treatment of bovine mastitis relies primarily on antimicrobial agents; however, their efficacy against environmental Gram-negative pathogens, particularly *E. coli*, remains limited, posing a major challenge for effective disease control and treatment [4,5]. Moreover, the long-term and widespread use of antimicrobial agents has accelerated the emergence and dissemination of antimicrobial resistance among environmental pathogens. In particular, the increasing resistance of *E. coli* to first-line antimicrobial agents has further complicated the clinical management of bovine mastitis [6].

The current bottleneck in the diagnosis and treatment of bovine mastitis caused by *Escherichia* spp. infection lies in the difficulty of achieving effective bacterial clearance while limiting excessive inflammatory injury [7,8]. Clinical high-throughput genomic studies have revealed that the coexistence of antimicrobial resistance and virulence genes enables epidemic strains to survive and disseminate more effectively under antibiotic selective pressure [9]. Consequently, these strains often trigger enhanced release of pro-inflammatory LPS during infection and treatment [10,11]. Current β-lactam antibiotics inevitably trigger massive LPS release when they kill bacteria by disrupting the bacterial cell wall [12]. The increased endotoxin burden markedly activates the host immune response and disrupts redox homeostasis [8,12,13]. Persistent inflammatory injury subsequently damages the mammary epithelium and ultimately results in an irreversible decline in lactation function [8,12,13]. Therefore, developing a non-antibiotic alternative strategy that can achieve both effective bacterial eradication and endotoxin neutralization is essential for improving the treatment of bovine mastitis caused by multidrug-resistant *E. coli*.

The bovine mammary gland has evolved multiple innate immune strategies to combat invading pathogens [14,15]. One key mechanism is nutritional immunity, whereby it limits iron availability by increasing the production of transferrin and lactoferrin, which bind ferric ions (Fe^3+^) with high affinity [14,15]. This strategy effectively limits extracellular bacterial growth and proliferation [14]. Previous studies have shown that nutritional immunity can also exert antibacterial effects through the formation of dinitrosyl iron complexes (DNICs) with nitric oxide (NO), thereby depriving Streptococcus pyogenes of available iron [16]. This finding provides a rationale for targeting bacterial iron metabolism in the development of therapies against extensively drug-resistant pathogens. Gallium ion (Ga^3+^) is considered a biochemical mimetic of Fe^3+^, due to its similar ionic radius, ionization potential, and electronic configuration [17,18]. In addition, Ga^3+^ is redox-inert and can interfere with bacterial oxidative phosphorylation and block the respiratory chain by competitively occupying iron-binding sites [19,20,21]. Gallium nitrate, the main donor form of Ga^3+^, significantly inhibits bacterial antioxidant activity by replacing iron in various metalloproteins [20,22,23]. It also downregulates the expression of key antioxidant enzyme genes, including *sodB*, *sodC*, *katE,* and *katG* [20,22,23]. This unique iron metabolism-targeting mechanism is distinct from the bactericidal pathways of metals such as silver and copper, which rely on inducing reactive oxygen species (ROS) or functional group reactions, and can effectively reduce the risk of bacterial drug resistance [20]. Existing studies have used gallium nitrate as an adjuvant to colistin to enhance its antibacterial activity and induce ROS accumulation to reduce the antioxidant activity of bacteria (*Klebsiella pneumoniae*) [20,23,24].

In the intricate physiological environment of the bovine mammary gland, a singular metabolic intervention may be insufficient to completely eradicate high loads of extensively drug-resistant (XDR) bacterial strains [2,6,25]. Because of the complex infection and inflammatory microenvironment associated with mastitis, antimicrobial peptides are ideal candidates for combination with metal ions due to their unique antibacterial mechanism and low propensity to induce bacterial drug resistance [2,26,27]. Thanatin, a host-derived antimicrobial peptide, has a high-affinity specific LPS-binding capacity [28,29]. It disrupts the integrity of the bacterial membrane structure by competitively displacing divalent cations in the outer membrane and significantly increases the permeability of the outer and inner membranes of Gram-negative bacteria [30,31]. More importantly, thanatin effectively neutralizes and reduces the biological activity of LPS, thereby alleviating its mediated inflammatory response [32,33].

Existing studies have confirmed that the combination of membrane-damaging agents and metabolism-interfering agents has favorable antibacterial potential. Moreira et al. demonstrated a strong synergistic antibacterial effect between Lynronne-1 (Lyn 1) and EDTA [34]. Their combined efficacy was significantly greater than that of either agent alone [34]. This combination also showed potential for development as an antibiotic-free therapeutic formulation [34]. However, for dairy-derived XDR strains, the molecular mechanisms of such combination regimens remain poorly understood within the complex mammary microenvironment, where both the milk matrix and host inflammatory responses may affect therapeutic efficacy [34].

In this study, clinical multidrug-resistant *E. coli* isolates obtained from bovine mastitis were investigated to address the dual challenges of antimicrobial resistance and LPS-induced inflammatory injury. The overall goal of this study was to characterize the genomic features of multidrug-resistant *E. coli*, identify potential therapeutic targets, and develop a precision therapeutic strategy guided by pathogen genomic characteristics. To achieve this goal, whole-genome sequencing was performed on the clinical multidrug-resistant isolates to comprehensively characterize their genomic features, antimicrobial resistance determinants, and virulence-associated genes. Based on the enrichment of siderophore-associated virulence genes identified through genomic analysis, a combination therapy consisting of Thanatin and gallium nitrate was developed. The therapeutic efficacy of this combination and its ability to alleviate LPS-induced inflammatory injury were subsequently evaluated in a murine mastitis model. Collectively, these findings provide a theoretical basis for the development of non-antibiotic therapeutic strategies against bovine mastitis caused by multidrug-resistant *E. coli* and offer new insights into the development of therapeutic approaches guided by pathogen genomic characteristics.

## 2. Materials and Methods

### 2.1. Clinical Milk Samples and Conventional Microbiological Analysis

Milk samples were collected aseptically from individual quarters of dairy cows with clinical mastitis during routine veterinary visits to commercial dairy farms. Clinical mastitis was diagnosed according to the guidelines of the International Dairy Federation [35]. Prior to sampling, teat ends were cleaned and disinfected, and the first few streams of milk were discarded. Foremilk samples were then collected into sterile tubes and transported to the laboratory on ice.

Upon arrival, 1 mL of each milk sample was inoculated into 9 mL of Brain Heart Infusion (BHI) broth and incubated at 37 °C with shaking (180 rpm) for 18–24 h. The enriched cultures were streaked onto Eosin Methylene Blue (EMB) agar for the selective isolation of *E. coli*. Additional media, including Columbia blood agar supplemented with 5% sheep blood, KF Streptococcus agar, and Mannitol Salt agar, were used for preliminary differentiation of mastitis-associated bacteria.

After incubation at 37 °C for 18–24 h, colonies exhibiting a characteristic metallic green sheen on EMB agar were selected and further purified in fresh BHI broth. Purified isolates were preserved in BHI broth containing 25% glycerol and stored at −80 °C for subsequent analyses.

### 2.2. Antimicrobial Susceptibility Testing

Antimicrobial susceptibility testing of *E. coli* isolates was performed using the broth microdilution method according to the Clinical and Laboratory Standards Institute (CLSI) guidelines [36]. Minimum inhibitory concentrations were determined in 96-well microtiter plates using cation-adjusted Mueller–Hinton broth (CAMHB; Solarbio Science & Technology Co., Ltd., Beijing, China).

The following antimicrobial agents were tested: amoxicillin (0.25–128 μg/mL), ampicillin (0.25–128 μg/mL), gentamicin (0.25–128 μg/mL), tetracycline (0.25–128 μg/mL), doxycycline (0.25–128 μg/mL), ciprofloxacin (0.25–128 μg/mL), polymyxin B (0.25–128 μg/mL), florfenicol (0.25–128 μg/mL), and sulfisoxazole (1–512 μg/mL).

Positive and negative controls were included in each assay, and *E. coli* ATCC 25922 was used as the quality control strain. MIC values were interpreted according to CLSI clinical breakpoints. Isolates resistant to three or more antimicrobial classes were defined as MDR.

### 2.3. Whole-Genome Sequencing and Genome Assembly

Multidrug-resistant *E. coli* isolates were cultured overnight in BHI broth at 37 °C. Genomic DNA was extracted using a bacterial genomic DNA extraction kit (Biorigin, Beijing, China) according to the manufacturer’s instructions. Qualified DNA samples were submitted to Tsingke Biotechnology Co., Ltd. (Beijing, China) for whole-genome sequencing using the Illumina NovaSeq platform with a paired-end 150 bp sequencing strategy.

Raw sequencing reads were processed for quality control by removing adapter sequences, reads with ≥10% ambiguous bases (N), and low-quality reads. The clean reads were de novo assembled using a combination of SOAPdenovo, SPAdes, and ABySS. The resulting assemblies were integrated using CISA, followed by gap filling with GapCloser. Only contigs ≥ 500 bp were retained for subsequent analysis. Open reading frames were predicted using GeneMarkS (v4.17). To identify virulence factors and antimicrobial resistance genes, the predicted protein sequences were aligned against the Virulence Factor Database and the Comprehensive Antibiotic Resistance Database using the Basic Local Alignment Search Tool (BLAST). A positive hit was defined with an E-value < 10^−5^, minimum sequence identity of 90%, and sequence coverage of 90%.

### 2.4. Phylogenetic Analysis

The 16S rRNA gene sequences of the isolates were compared against the National Center for Biotechnology Information (NCBI) database using the BLAST algorithm to identify and select representative reference sequences with high homology. These obtained sequences, along with the reference sequences, were imported into MEGA 11 software and aligned using the MUSCLE method. Subsequently, a phylogenetic tree was constructed using the Neighbor-Joining method, with genetic distances calculated via the Kimura 2-parameter model. Gaps and missing data were treated using the pairwise deletion option. The reliability of the tree branches was evaluated by bootstrap analysis with 1000 replicates. The resulting phylogenetic tree was exported and uploaded to the Interactive Tree of Life online platform for visualization and refinement.

### 2.5. Animals and Study Design

#### 2.5.1. Statement of Ethics

This study was conducted in compliance with the guidelines of the Beijing Municipality on the Review of Welfare and Ethics of Laboratory Animals. All animal protocols were approved by the Beijing Municipality Administration Office of Laboratory Animals (BAOLA) and the Animal Ethics Committee of Beijing University of Agriculture (Protocol number: BUA612512151).

#### 2.5.2. Laboratory Animals

To establish an *E. coli*-induced mastitis rat model, a total of 80 specific pathogen-free Sprague-Dawley rats were purchased from Beijing Sibeibo Biotechnology Co., Ltd. (Beijing, China), including 60 nulliparous female rats weighing 200–220 g and 20 male rats weighing 320–400 g. Following a one-week acclimation period with ad libitum access to food and water, the rats were housed at a mating ratio of three females to one male per cage. Pregnant females were subsequently housed individually until parturition.

#### 2.5.3. Rat Model Establishment

At 72 h postpartum, the 60 lactating female rats were randomly divided into 6 groups with 10 rats per group. These six groups were the uninfected control group, the untreated infection group, the Ga^3+^ treatment group, the Tn treatment group, the Ga^3+^ plus Tn combination treatment group, and the ceftiofur positive control group. Two hours before model establishment, pups were separated from the dams to allow milk accumulation in the mammary glands. Intramammary inoculation was performed according to the method described. After anesthesia with isoflurane, the nipple areas of the left and right 4th pairs of abdominal mammary glands were disinfected with 75% ethanol. A 31-gauge needle attached to a 0.5 mL syringe was gently inserted into the lactiferous duct. Each mammary gland in the infection group was slowly injected with 100 μL of *E. coli* suspension at a concentration of 1 × 10^7^ CFU/mL. Rats injected with sterile phosphate-buffered saline (PBS) only served as the uninfected control group.

#### 2.5.4. Treatment

Drug administration was initiated 1 h after model establishment, and all treatments were administered through the mammary duct. The gallium nitrate (Ga(NO_3_)_3_) treatment group received 10 mg/kg gallium nitrate. This dosage was selected based on a previously established rat model of *E. coli*-induced mastitis, in which the same dose was demonstrated to provide effective therapeutic efficacy and has been widely adopted as a positive control in similar studies [37]. The combination treatment group received 5 mg/kg thanatin plus 10 mg/kg gallium nitrate. The CEF positive control group received 10 mg/kg ceftiofur. Twelve hours after the first dose, the uninfected control group, the untreated infection (model) group, and the ceftiofur positive control group were injected with an equal volume of PBS. Mammary tissues were collected under sterile conditions; a portion of the samples was stored at −80 °C for further analyses, while the remaining mammary tissues were fixed in 4% paraformaldehyde for subsequent hematoxylin and eosin (H&E) staining.

#### 2.5.5. Preparation of Mammary Tissue

Mammary tissues were aseptically collected, weighed, and homogenized with sterile PBS (1:9, *w*/*v*) on ice. An aliquot was used for bacteria count, and the remaining homogenate was centrifuged at 3000 rpm for 20 min at 4 °C. The fat was removed, and the supernatant was collected and stored at −80 °C until analyzed.

#### 2.5.6. Bacterial Load in the Mammary Gland

Homogenates of the mammary gland were serially diluted in PBS and then plated on Luria–Bertani (LB) agar. After incubation at 37 °C for 18–24 h, bacterial colonies were counted, and the number of CFU per gram of mammary tissue was calculated.

#### 2.5.7. Enzyme-Linked Immunosorbent Assay (ELISA)

The levels of LPS in the mammary tissues were determined using a Limulus amebocyte lysate (LAL) assay kit. The concentrations of pro-inflammatory cytokines (TNF-α, IL-1β, and IL-6) and the activity of myeloperoxidase (MPO) were measured using specific commercial ELISA kits (Shanghai Enzyme-linked Biotechnology Co., Ltd., Shanghai, China). All measurement procedures were performed strictly according to the manufacturer’s instructions.

#### 2.5.8. Histopathological Observation

The mammary gland tissues fixed in 4% paraformaldehyde were dehydrated through a series of graded alcohols, cleared in xylene, and embedded in paraffin wax. The paraffin blocks were sectioned and stained with H&E. The scoring of histopathological changes was performed blindly based on a previously described method [38,39], and the detailed scoring criteria are shown in Table S3.

### 2.6. Statistical Analyses

All data are expressed as means ± standard error of the mean (Means ± SEM). Statistical analyses and graphing were performed using GraphPad Prism software (version 10.0; GraphPad Software, Boston, MA, USA). Comparisons among multiple groups were analyzed using one-way analysis of variance followed by Tukey’s multiple comparison test. Statistical significance was declared at *p* < 0.05. Final image assembly and processing were performed using Adobe Illustrator 2020.

## 3. Results

### 3.1. Isolation and Phylogenetic Characterization of Escherichia spp. from Bovine Mastitis Milk

A total of 93 bacterial isolates were obtained from milk samples of bovine mastitis. Among them, *E. coli* was the predominant species with 68 isolates, accounting for 73.11%, followed by *E. fergusonii* with 25 isolates, accounting for 26.88%.

To further elucidate the genetic relationships among the isolates, a phylogenetic tree was constructed based on 16S rRNA gene sequences using the neighbor-joining method. As shown in Figure S1, all isolates clustered within the genus *Escherichia* and formed two distinct clades corresponding to *E. coli* and *E. fergusonii*. Most isolates were grouped within the *E. coli* clade, exhibiting a high degree of genetic homogeneity, whereas *E. fergusonii* isolates formed a separate but closely related clade.

In addition, no apparent interspecies clustering was observed, indicating clear discrimination at the species level. The relatively short branch lengths within each clade suggest limited genetic divergence among the isolates, which may reflect a common environmental origin or potential epidemiological relatedness.

### 3.2. Antimicrobial Resistance and Genomic Characteristics of E. coli

**Figure 1:**
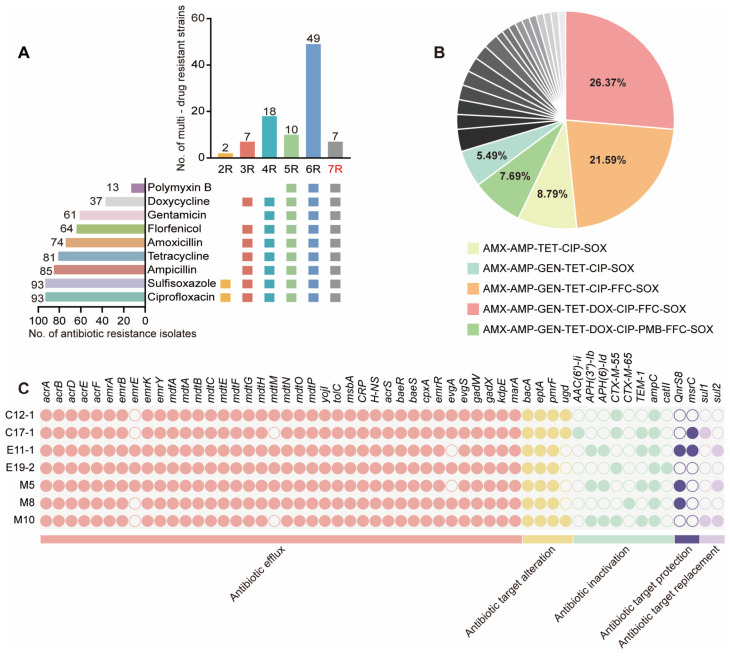
Antimicrobial resistance profiles of multidrug-resistant *E. coli*. (**A**) Antimicrobial resistance frequencies and multidrug resistance (MDR) profiles of *Escherichia* spp. isolates. The matrix plot illustrates the co-occurrence of resistance phenotypes. (**B**) Proportional distribution of phenotypic MDR profiles among the isolates. (**C**) Heatmap illustrating the distribution of virulence and antibiotic resistance genes in 7 selected MDR *E. coli* strains. Genes are categorized by their primary resistance mechanisms, including antibiotic efflux, inactivation, target protection, target alteration, and target replacement.

Antimicrobial susceptibility testing and antimicrobial resistance gene (ARG) analysis were performed on 93 *Escherichia* spp. isolates derived from bovine mastitis. Detailed MIC values for all tested antimicrobial agents are provided in Supplementary Table S1, and the corresponding antimicrobial susceptibility profiles are summarized in Supplementary Table S2. Among the 93 isolates, the multidrug resistance rate was 97.85% (91/93). As shown in Figure 1A, the susceptibility testing results for nine antibiotics revealed that all isolates (100%, 93/93) were resistant to ciprofloxacin and sulfisoxazole. Furthermore, extremely high resistance rates were observed for ampicillin (91.40%, 85/93), tetracycline (87.09%, 81/93), and amoxicillin (79.57%, 74/93). The isolates also exhibited high resistance to gentamicin (65.59%, 61/93) and florfenicol (68.81%, 64/93), while the resistance rate for doxycycline (39.78%, 37/93) was slightly lower, and polymyxin B showed the lowest resistance rate at 13.97% (13/93). Strains resistant to six classes of antibiotics were the most frequent (*n* = 49), followed by those resistant to three (*n* = 7), five (*n* = 10), and four classes (*n* = 18). Strains resistant to seven classes of antibiotics (7 isolates) exhibited a broad resistance spectrum and accounted for a considerable proportion.

Figure 1B further illustrates the proportional distribution of MDR phenotypes. The most prevalent MDR phenotype was AMX-AMP-GEN-TET-DOX-CIP-FFC-SOX (26.37%), a combination covering eight antibiotics across six classes, including penicillins, gentamicin, tetracyclines, doxycycline, quinolones, florfenicol, and sulfonamides. This was followed by AMX-AMP-GEN-TET-CIP-PMB-FFC-SOX (21.59%), reflecting the widespread co-resistance characteristics of the isolates.

Widespread presence of efflux pump determinants suggests that these systems may contribute significantly to the broad-spectrum resistance phenotype observed in these isolates. Additionally, most strains harbored target modification genes such as *bacA*, *eptA*, *pmrF*, and *ugd*. They also carried antibiotic inactivation genes, including aminoglycoside modifying enzyme groups APH (3″)-lb and APH (6)-ld, as well as extended-spectrum beta-lactamase genes such as *CTX-M-55*, *CTX-M-65*, and *TEM-1*. Target protection and replacement genes, such as the quinolone resistance gene *QnrS8*, were also identified. These results confirm the potential for the transmission of multidrug resistance among these pathogens.

### 3.3. Virulence Factor Profile Analysis of E. coli Resistant to Seven Classes of Antibiotics

**Figure 2:**
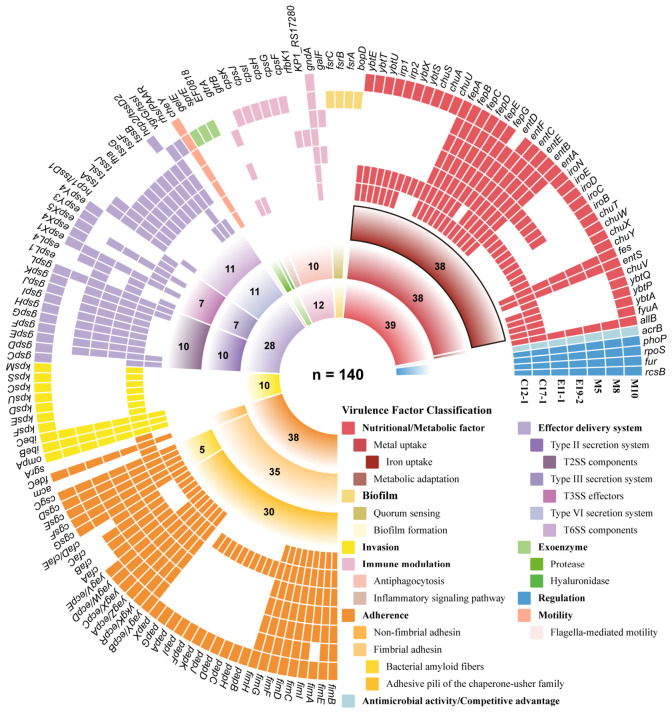
Functional classification of 140 virulence genes in multidrug-resistant *E. coli*. The circular plot displays the presence or absence of virulence-associated genes across functional categories, including nutritional/metabolic factors, biofilm formation, invasion, immune modulation, adherence, effector delivery systems, exoenzymes, regulation, motility, and antimicrobial activity.

Following the determination of the resistance characteristics of these seven strains, this study further conducted an in-depth analysis of their virulence-related genes (Figure 2). Gene prediction results revealed that a total of 140 virulence-related genes were detected among the seven strains resistant to seven classes of antibiotics, encompassing 10 functional categories: nutrition and metabolism, biofilm formation, adhesion, invasion, immune regulation, effector delivery systems, extracellular enzymes, regulation, motility, and antimicrobial competition.

Nutritional and metabolic factors represented the functional category with the highest proportion among the seven strains. Within this category, iron uptake-related genes were particularly abundant, with a total of 38 genes identified. Iron uptake systems, such as the *chu*, *ent*, *fep*, and *ybt* families, were highly conserved and complete across all strains. This demonstrates that these strains possess robust survival and competitive capabilities within the low-iron environment of the bovine mammary gland.

A total of 38 genes related to adhesion were detected, among which the chaperone-usher fimbriae families (*fim* and *pap*) were the most prominent. Adhesion factors are the critical links for pathogens to colonize mammary tissue and initiate infection. The effector delivery system category comprised 28 genes, providing complete coverage of Type II (T2SS), Type III (T3SS), and Type VI (T6SS) secretion systems. Specifically, the prevalence of the T6SS core component genes *vgrG* and *hcp* suggests that these strains possess potent interspecies competitiveness and the ability to attack host cells. Despite being isolated from different samples, the distribution of virulence genes across the seven strains exhibited a high degree of homology and consistency.

### 3.4. Therapeutic Effects of Tn Combined with Ga^3+^ on E. coli-Induced Mastitis in Rats

**Figure 3:**
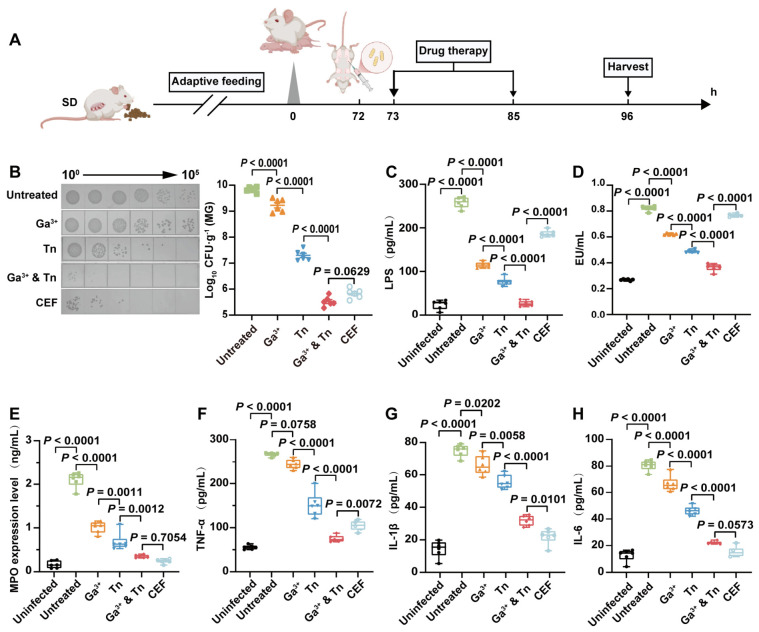
Therapeutic efficacy of gallium nitrate and Tn against *E.coli*-induced mastitis in vivo. Sprague-Dawley rats were intramammarily inoculated with *E. coli* (1 × 10^7^ cfu) and treated with Ga^3+^ (10 mg/kg), Tn (5 mg/kg), their combination, or CEF at 2 h post-infection. (**A**) Schematic timeline of the experimental design. (**B**) Bacterial load (log_10_ cfu/g) in mammary gland tissues at 24 h post-treatment. (**C**) LPS concentration and (**D**) endotoxin activity in mammary tissues determined by the LAL assay. (**E**) MPO expression levels indicate neutrophil infiltration. (**F**–**H**) Concentrations of pro-inflammatory cytokines TNF-α (**F**), IL-1β (**G**), and IL-6 (**H**) in mammary tissues.

To evaluate the in vivo efficacy, a rat model of *E. coli* mastitis was established, as outlined in Figure 3A. At 24 h post-infection, the bacterial load in the mammary tissues of the Untreated group was high, reaching approximately 10^9^ CFU/g. While monotherapy with either Ga^3+^ or Tn reduced the bacterial count to some extent, the Ga^3+^ and Tn combination group exhibited the most significant bactericidal effect, with the bacterial load dropping to approximately 10^5.5^ CFU/g (*p* < 0.0001). The combination treatment exhibited significantly greater antibacterial efficacy than either monotherapy and achieved a bactericidal effect comparable to that of the clinical positive control, CEF. Consistent with the reduction in bacterial burden, the LPS concentration and endotoxin activity in mammary tissues were also markedly decreased in the combination group compared with the untreated group (Figure 3C,D). Although CEF effectively reduced the bacterial burden, LPS concentrations remained relatively high (approximately 200 pg/mL).

Compared with the Uninfected group, the expression levels of pro-inflammatory markers (TNF-α, IL-1β, and IL-6) and the inflammatory enzyme MPO were significantly elevated in the mammary tissue homogenates of the Untreated group. However, the combination of Tn and Ga^3+^ significantly downregulated the expression of these inflammatory factors, showing a significant difference compared to the monotherapy groups (*p* < 0.0001). Particularly in terms of TNF-α and IL-6 inhibition, the combination group demonstrated a trend that was superior to or at least consistent with the CEF group. Furthermore, MPO levels in the combination group were significantly lower than those in the monotherapy and Untreated groups, indicating that the treatment effectively reduced the infiltration and accumulation of inflammatory cells within the mammary tissue.

### 3.5. Protective Effects of Tn Combined with Ga^3+^ on Mammary Histopathology

The study observed diffuse hyperemia and edema, massive neutrophil infiltration, and acinar necrosis in the mammary tissues of rats. These symptoms were primarily attributed to the rapid proliferation of *E. coli* and the subsequent triggering of a severe inflammatory cascade by the massive release of LPS. To objectively evaluate the degree of injury, pathological scoring was performed based on the extent of hyperemia, edema, acinar necrosis, and neutrophil infiltration (Table S3).

**Figure 4:**
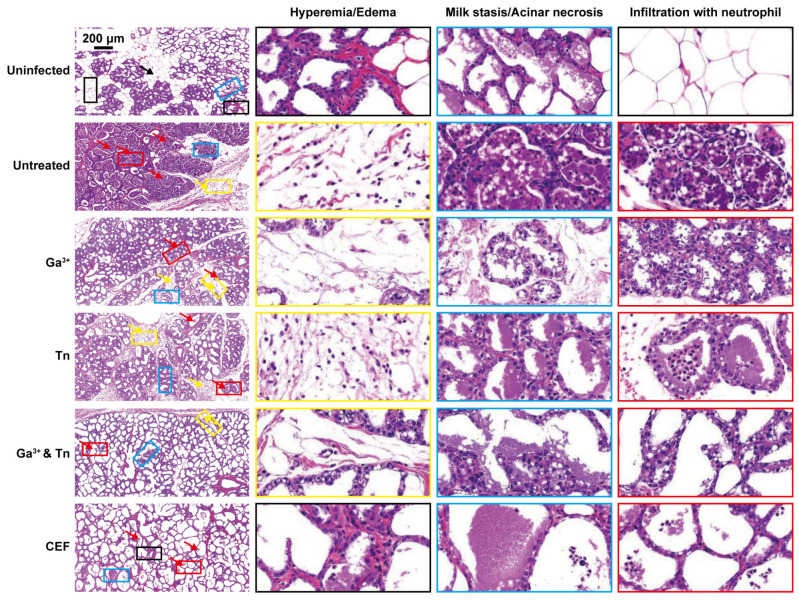
Histopathological analysis of *E. coli*-induced mastitis after treatment. Ga^3+^ and Tn combination treatment. Representative hematoxylin and eosin-stained tissue sections from uninfected, untreated, Ga^3+^, Tn, Ga^3+^, and Tn, and CEF groups are shown. Boxed regions highlight specific pathological features: normal tissue architecture (black boxes), congestion and edema (yellow boxes), milk stasis and alveolar necrosis (blue boxes), and severe neutrophil infiltration (red boxes). Red labeling arrows point to severe neutrophil infiltration. Black labeling arrows indicate normal tissue architecture. Yellow labeling arrows point to congestion and edema.

The Uninfected group displayed healthy mammary tissue morphology, with clear and tightly arranged acinar structures, intact acinar epithelial cells, and no interstitial hyperemia or inflammatory cell infiltration. In contrast, the Untreated group exhibited severe tissue damage (Table 1 and Figure 4). While monotherapy with either Ga^3+^ or Tn attenuated the progression of the disease to some extent, significant inflammatory infiltration and structural damage to the acini remained visible. Compared to the Untreated group, single-agent treatments provided limited protection; persistent clusters of inflammatory cells and localized acinar collapse were still observed in the sections, indicating that a single agent is insufficient to fully arrest the acute pathological progression induced by multidrug-resistant *E. coli*.

The Ga^3+^ and Tn combination group demonstrated superior tissue-protective effects compared to the monotherapy groups. Histopathological examination revealed that the mammary acinar structures were well preserved, closely resembling those of the Uninfected group, with a significant resolution of hyperemia and edema, and minimal neutrophil infiltration. The histopathological improvement was comparable to that observed in the ceftiofur-treated group. Moreover, the combination treatment showed greater preservation of acinar architecture, including improved acinar lumen integrity and reduced necrotic areas. These findings indicate that the combination of Tn and Ga^3+^ not only effectively eliminates bacterial pathogens but also protects mammary tissue integrity by reducing inflammation-associated tissue injury.

## 4. Discussion

### 4.1. Contextual Relevance

Currently, the global dairy industry is facing a severe challenge due to the escalating threat of antimicrobial resistance (AMR) [40,41,42]. In intensive farming systems, antibiotics are extensively utilized for both treating infections and disease prevention [43]. This prolonged antibiotic pressure, combined with host immune pressure, has been shown to significantly exacerbate bacterial colonization [44,45]. These factors collectively amplify the frequency of genetic exchange, thereby driving the horizontal gene transfer of resistance plasmids and virulence genes [46,47].

In this study, we delineated the highly complex AMR and virulence profiles of clinically isolated *E. coli* via WGS. The results showed that the 7-antibiotic-resistant *E. coli* strains identified in this study harbored not only gene clusters involved in five major AMR mechanisms (Figure 1) but also up to 140 virulence factors (Figure 2). Among these, the significant enrichment of genes associated with the iron uptake system revealed the robust colonization and survival capacity of this bacterial population in the iron-restricted microenvironment of the mammary gland [8,48,49]. This combined feature of AMR and virulence not only objectively confirms the complexity of clinical treatment but also provides precise targets for the development of targeted non-antibiotic strategies.

In response to the above genomic characteristics, we developed a combination therapy of gallium nitrate and the antimicrobial peptide thanatin. The core advantage of this regimen lies in its dual-hit mechanism of metabolic interference and membrane barrier disruption. Multidrug-resistant *E. coli* strains possess highly developed siderophore channels, enabling Ga^3+^, an iron mimetic, to efficiently enter the bacterial interior via the “Trojan horse” effect [20,22,30]. Owing to its non-reducible chemical properties, Ga^3+^ competitively occupies the iron-binding sites in cytochromes and other metalloproteins, thereby endogenously blocking the bacterial respiratory electron transport chain [17,20]. Meanwhile, thanatin disrupts outer membrane integrity by displacing cations from the outer membrane, which not only exerts a direct bactericidal effect but also accelerates bacterial uptake of Ga^3+^ by increasing membrane permeability [30,47,50]. These complementary mechanisms may explain why the combination treatment exhibited greater antibacterial activity than either monotherapy against XDR strains harboring multiple AMR mechanisms.

Another key finding of this study is the effective regulation of LPS-induced injury by the combination regimen. Consistent with previous reports, β-lactam antibiotics can induce endotoxin release during bacterial lysis, thereby aggravating host inflammatory responses [12,51,52,53,54]. The experimental results confirmed that the LPS concentration and key pro-inflammatory cytokines (TNF-α, IL-1β, IL-6) in the mammary tissue of the combination group were significantly decreased (Figure 3). The unique LPS-neutralizing activity of thanatin and the inhibitory effect of Ga^3+^ on bacterial antioxidant enzyme activity are complementary, which limits the pathogen load from the source and blocks the inflammatory cascade [31,50].

Histopathological evaluation further intuitively demonstrated the advantage of this regimen in maintaining the integrity of the acinar structure (Figure 4 and Table 1) [55,56]. Although the combination treatment achieved antibacterial efficacy comparable to that of CEF, it provided greater protection against LPS-induced inflammation and mammary tissue injury, as evidenced by reduced acinar edema and neutrophil infiltration. This protective effect on tissue ultrastructure has remarkable clinical significance for maintaining the lactation function and prolonging the productive lifespan of dairy cows [57,58].

Taken together, this study reveals the grim status of the co-evolution of antimicrobial resistance and virulence in milk-derived *E. coli* from dairy cows and confirms the great potential of the combination of gallium nitrate and thanatin against extensively drug-resistant bacterial infections via multi-target mechanisms. This study not only provides a theoretical basis for the clinical prevention and control of drug-resistant mastitis but also offers new insights into the development of combination therapeutic strategies targeting bacterial iron metabolism and endotoxin neutralization.

### 4.2. Future Research Directions

The present study identified a significant enrichment of iron acquisition-related virulence genes in multidrug-resistant *E. coli* isolated from bovine mastitis through whole-genome sequencing. Based on these genomic characteristics, we developed a targeted therapeutic strategy combining thanatin with gallium nitrate and demonstrated its favorable antibacterial and anti-inflammatory efficacy in a rat model of mastitis. However, substantial differences remain between the rat model and the bovine mammary gland in terms of anatomical structure, milk composition, immune microenvironment, and disease pathophysiology. Therefore, the therapeutic potential of this combination should be further validated in bovine mastitis models and naturally infected dairy cows. Future studies should systematically evaluate its ability to achieve sustained bacterial clearance, control inflammation, and promote mammary tissue repair, while optimizing the dosage, dosing frequency, and route of administration to facilitate clinical application in dairy cattle [59,60].

Furthermore, this study proposes a genome-guided strategy for developing targeted therapies against multidrug-resistant pathogens. Nevertheless, the distribution of iron metabolism-related virulence factors among *E. coli* strains with different serotypes, geographic origins, and host sources, as well as their association with therapeutic efficacy, remains to be elucidated. Future multicenter genomic studies involving a larger collection of clinical isolates are warranted to validate these findings. In addition, the molecular basis underlying the combined action of thanatin and Ga^3+^ should be further investigated to clarify their effects on bacterial iron metabolism, virulence regulation, and host inflammatory responses, thereby providing mechanistic support for genome-guided precision antimicrobial therapy. Future studies should also include comprehensive pharmacokinetic analyses, safety evaluations, assessments of milk residues and withdrawal periods, as well as formulation optimization and scale-up manufacturing to facilitate the clinical translation and commercialization of this therapeutic strategy [59,60].

## 5. Conclusions

This study integrated whole-genome sequencing with in vivo therapeutic evaluation to systematically characterize the antimicrobial resistance profiles, virulence spectrum, and potential pathogenic mechanisms of multidrug-resistant *E. coli* clinical isolates from bovine mastitis. Genomic analysis revealed that the strains carried multiple resistance genes and a rich repertoire of virulence factors, with significant enrichment of iron acquisition-related virulence genes. This highlights the iron metabolism system as a critical biological basis for the pathogen’s ability to maintain infection and cause disease, providing a theoretical foundation for the design of subsequent treatment strategies.

Based on these findings, we developed a non-antibiotic therapeutic strategy combining Thanatin and gallium nitrate, which demonstrated promising therapeutic efficacy in a mouse mastitis model. The combination regimen effectively alleviated inflammatory damage in mammary gland tissue, reduced LPS levels and inflammatory cytokine expression, and improved histopathological lesions. It exhibited superior overall therapeutic advantages compared to monotherapy. This study provides a potential non-antibiotic intervention strategy for bovine mastitis caused by resistant *E. coli* and offers new theoretical support for the development of precision treatment strategies guided by pathogen genomic characteristics.

## Figures and Tables

**Table 1:** Histopathological Scores of Mammary Gland Tissues Across Experimental Groups.

Group	Hyperemia/Edema	Milk Stasis/Acinar Necrosis	Infiltration with Neutrophil	Inflammation Score
Uninfected	0 ± 0	1 ± 0	0.33 ± 0.58	1.33 ± 0.58 ****
Untreated	3 ± 0	3 ± 0	5 ± 0	11 ± 0
Ga^3+^	1.67 ± 1.15	2.33 ± 0.58	4.67 ± 0.58	8.67 ± 0.58 ***
Tn	2.33 ± 1.15	1.67 ± 0.58	2.67 ± 0.58	6.67 ± 0.58 ****
Ga^3+^ and Tn	0.67 ± 0.58	1 ± 0	1.67 ± 0.58	3.33 ± 0.58 ****
CEF	0.67 ± 0.58	1.67 ± 0.58	2 ± 1	4.33 ± 0.58 ****

Note: Data are presented as mean ± SEM. *** *p* < 0.001, **** *p* < 0.0001 compared with the Untreated group.

## Data Availability

The datasets presented and generated in this work are available on request to the corresponding author. The whole-genome sequencing data generated in this study have been deposited in the NCBI BioProject database under accession number PRJNA1444032, available at: https://www.ncbi.nlm.nih.gov/bioproject/PRJNA1444032 (accessed on 2 April 2026).

## Supplementary Materials

The following supporting information can be downloaded at: https://www.mdpi.com/article/10.3390/microorganisms14071538/s1. Figure S1: Phylogenetic tree of *Escherichia* spp. isolates from bovine mastitis based on 16S rRNA gene sequences; Table S1: Minimum inhibitory concentrations (MICs) of 93 *Escherichia* spp. isolates against the tested antimicrobial agents; Table S2: Antibiotic Sensitivity Profiles of *Escherichia* spp. (*n* = 93) Isolated from Milk Samples of Bovine Cows; Table S3: Inflammatory Scoring Criteria for Mammary Gland Tissues.
