# Supplementary material for: Genomic Characterization of Multidrug-Resistant Escherichia coli from Bovine Mastitis and Therapeutic Evaluation of Thanatin Combined with Gallium Nitrate
Source: Microorganisms. 2026 Jul 14;14(7):1538. doi: 10.3390/microorganisms14071538 (PMC13413798; doi:10.3390/microorganisms14071538)
Supplement: Supplementary file 1 [file microorganisms-14-01538-s001.zip › microorganisms-4383919-supplementary.pdf]

Supplementary Information (SI)

Genomic Characterization of Multidrug-Resistant *Escherichia coli* from Bovine Mastitis and Therapeutic Evaluation of Thanatin Combined with Gallium Nitrate

This file includes:

A.Supplementary tables

Table S1 to S3

B.Supplementary figures

Figure S1

Table S1. Minimum inhibitory concentrations (MICs) of 93 *Escherichia* spp. isolates against the tested antimicrobial agents.

Strains	MIC (μg / mL)								
	**AMX**	**AMP**	**GEN**	**TET**	**DOX**	**CIP**	**PMB**	**FFC**	**SOX**
E. coli E3-3	4	<	4	<	<	64	<	4	>
E. Fergusonii E3-4	4	<	2	<	<	128	<	4	>
E. coli E10-2	4	<	8	<	<	4	<	16	>
E. coli E7-4	2	2	2	8	2	64	<	4	>
E. Fergusonii E5-3	4	<	4	16	<	4	<	4	>
E. Fergusonii E20-2	2	<	2	16	4	64	<	4	>
E. coli E7-1	>	>	2	<	<	2	<	4	>
E. Fergusonii E7-3	4	<	4	64	16	128	<	4	>
E. Fergusonii E16-3	4	<	2	64	16	128	<	4	>
E. Fergusonii E20-3	8	64	4	16	<	32	<	4	>
E. Fergusonii E19-4	16	32	4	32	4	64	<	4	>
E. Fergusonii E2-2	2	32	2	64	8	64	<	4	>
E. coli E1-4	4	64	4	2	2	2	<	16	>
E. Fergusonii E10-3	4	>	4	2	2	16	<	16	>
E. Fergusonii E1-3	128	64	8	64	8	128	<	4	>
E. coli E2-1	>	>	2	64	8	64	<	4	>
E. Fergusonii E2-3	32	32	4	32	4	64	<	4	>
E. Fergusonii E9-2	32	32	2	64	8	64	<	4	>
E. Fergusonii E12-3	128	64	4	32	4	64	<	4	>
E. Fergusonii E14-2	128	32	4	32	8	64	<	4	>
E. Fergusonii E16-4	>	64	2	64	8	64	<	4	>
E. Fergusonii E24-4	64	32	2	32	4	64	<	4	>

E. coli E7-2	>	>	64	<	<	8	<	4	>
E. coli E19-1	>	>	2	<	<	2	<	64	>
E. Fergusonii E21-3	2	<	4	64	16	128	<	16	>
E. coli E12-4	4	32	8	64	16	128	<	4	>
E. Fergusonii E19-3	4	32	4	64	16	64	<	4	>
E. coli E9-3	4	32	4	16	8	64	<	8	>
E. Fergusonii E21-4	4	32	2	32	8	64	<	8	>
E. coli E8-1	>	>	64	32	8	32	<	4	>
E. coli E17-3	>	>	64	64	8	128	<	4	>
E. coli E18-2	>	>	16	64	8	64	<	4	>
E. coli C2-2	>	>	16	64	8	64	<	4	>
E. coli C10-2	>	>	16	64	<	64	<	4	>
E. Fergusonii E18-3	4	32	8	64	16	64	64	4	>
E. Fergusonii E24-3	>	>	2	64	8	64	<	>	>
E. coli E14-1	>	128	64	8	8	32	<	>	>
E. coli E1-2	>	>	>	>	32	>	<	>	>
E. coli E3-1	>	>	>	>	128	>	<	>	>
E. coli E3-2	>	>	>	>	32	>	<	>	>
E. coli E4-1	>	>	128	128	32	>	<	>	>
E. coli E4-2-1	>	>	>	128	16	>	<	>	>
E. coli E5-1	>	>	>	>	64	>	<	>	>
E. coli E8-2	>	>	>	>	32	>	<	>	>
E. coli E10-1	>	>	>	>	64	>	<	128	>
E. coli E12-2	>	>	128	32	16	16	<	>	>
E. coli E13	>	>	>	>	32	>	<	>	>
E. coli E16-2	>	>	64	128	32	128	<	128	>

E. Fergusonii E18-4	>	>	>	64	16	64	<	>	>
E. Fergusonii E21-1	>	>	>	128	64	>	1	128	>
E. coli E22	>	>	64	128	32	128	<	>	>
E. coli E23-1	>	>	>	>	32	>	<	>	>
E. coli E23-2	>	>	>	>	32	>	<	>	>
E. coli E24-1	>	>	128	128	16	128	<	>	>
E. coli E24-2	>	>	128	>	32	128	<	>	>
E. coli C4	>	>	>	>	64	>	<	>	>
E. coli C8-1	>	>	64	128	32	128	<	>	>
E. coli C10-1	>	>	>	>	>	>	<	>	>
E. coli C12-2	>	>	>	64	64	>	<	>	>
E. coli C13	>	>	128	128	16	>	<	>	>
E. coli C23	>	>	>	>	32	>	<	>	>
E. coli E4-3	128	>	64	64	8	128	<	128	>
E. Fergusonii E5-2	>	>	>	32	4	64	<	16	>
E. Fergusonii E14-3	>	>	16	32	4	64	<	16	>
E. coli E15-1	>	>	128	64	8	32	<	>	>
E. coli E15-4	>	>	128	64	8	32	<	>	>
E. coli E16-1	>	>	64	32	4	64	<	64	>
E. coli E17-1	>	>	16	64	8	128	<	128	>
E. coli E21-2	>	>	64	32	4	32	<	64	>
E. coli C8-2	>	>	32	32	8	128	<	64	>
E. coli C17-2	>	>	64	64	8	64	<	16	>
E. coli E11-2	>	>	>	64	8	128	<	>	>
E. coli E11-3	>	>	128	64	8	32	<	>	>
E. coli E12-1	>	>	128	64	4	>	<	>	>

E. coli E15-2	>	>	>	64	8	32	<	>	>
E. coli E15-3	>	>	>	128	8	128	<	>	>
E. coli M19	>	>	64	64	8	>	<	>	>
E. coli C20-2	>	>	64	32	4	>	<	>	>
E. coli C21	>	>	32	64	8	64	<	>	>
E. coli C24-2	>	>	64	128	8	128	<	>	>
E. coli C7	>	>	16	32	8	8	<	>	>
E. coli E18-1	>	>	32	8	4	128	>	128	>
E. coli E1-1	>	>	128	8	4	>	16	128	>
E. coli E20-1	>	>	32	64	8	128	128	4	>
E. coli E9	>	>	8	32	4	128	128	128	>
E. coli E17-2	>	>	8	64	8	128	32	>	>
E. coli E11-1	>	>	>	64	64	>	>	>	>
E. coli E19-2	>	>	>	64	64	>	64	>	>
E. coli M5	>	>	>	128	64	>	>	>	>
E. coli M8	>	>	>	128	64	>	128	>	>
E. coli M10	>	>	>	64	64	>	>	>	>
E. coli C12-1	>	>	>	64	64	>	32	>	>
E. coli C17-1	>	>	>	64	64	>	>	>	>

Notes: Abbreviations of antimicrobial agents: AMX, amoxicillin; AMP, ampicillin; GEN, gentamicin; TET, tetracycline; DOX, doxycycline; PMB, polymyxin B; CIP, ciprofloxacin; LIC, lincomycin; FFC, florfenicol; SOX, sulfisoxazole; EM, erythromycin; MIC (minimum inhibitory concentration): the lowest concentration of each antimicrobial agent that inhibits visible bacterial growth. The symbol “<” represents the minimum test concentration of the corresponding drug, while “>” represents the maximum test concentration. Isolates were recovered from milk samples of cows with clinical mastitis, including *E. coli* and *E. fergusonii*.

Table S2. Antibiotic Sensitivity Profiles of *Escherichia* spp. (n = 93) Isolated from Milk Samples of Bovine Cows

Antibiotic used	***Escherichia* spp.**		
	Susceptible %	Intermediate %	Resistant %
Amoxicillin	18 (19.35)	1 (1.08)	74 (79.57)
Ampicillin	8 (8.6)		85 (91.40)
Gentamicin	27 (29.03)	5 (5.37)	61 (65.59)
Tetracycline	8 (8.6)	4 (4.30)	81 (87.09)
Doxycycline	26 (27.96)	30 (32.25)	37 (39.78)
Polymyxin B		80 (86.02)	13 (13.97)
Ciprofloxacin			93 (100)
Florfenicol	1 (1.07)	28 (30.1)	64 (68.81)
Sulfisoxazole			93 (100)

Table S3. Inflammatory Scoring Criteria for Mammary Gland Tissues

Feature	Description		Score
Hyperemia/Edema	Normal		0
	Mild		1
	Severe		3
Milk stasis/Acinar necrosis	Normal		0
	Mild		1
	Moderate		2
	Severe		3
Infiltration with neutrophil	0-1	Acinar or mammary gland neutrophil	0
	2-5	Acinar or mammary gland neutrophil	1
	6-10	Acinar or mammary gland neutrophil	2
	11-15	Acinar or mammary gland neutrophil	3
	16-20	Acinar or mammary gland neutrophil	4
	> 20	Acinar or mammary gland neutrophil	5

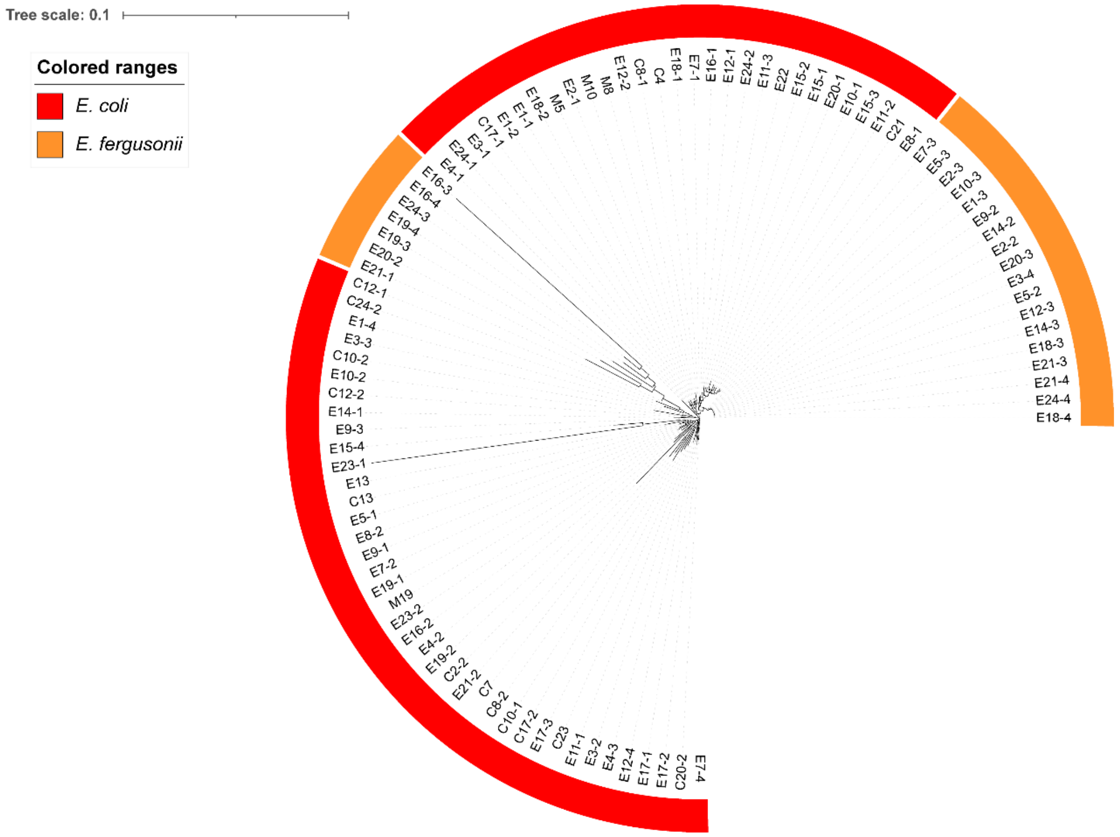

Figure S1. Phylogenetic tree of *Escherichia* spp. isolates from bovine mastitis based on 16S rRNA gene sequences. The phylogenetic tree was constructed using the neighbor-joining method in MEGA 11 software with 1000 bootstrap replicates. Red branches represent *E. coli* isolates, and orange branches represent *E. fergusonii* isolates. The scale bar indicates 0.1 nucleotide substitutions per site. All 93 isolates clustered within the genus *Escherichia* and formed two distinct clades corresponding to *E. coli* and *E. fergusonii*, respectively.
